# Metal-Free Elemental Selenium Quantum Dots: A Novel and Robust Fluorescent Nanoprobe for Cell Imaging and the Sensitive Detection of Cr(VI)

**DOI:** 10.3390/ma18092119

**Published:** 2025-05-05

**Authors:** Ziyi Gao, Jie Liao, Xia Li, Li Zhou

**Affiliations:** 1 Guangxi Colleges and Universities Key Laboratory of Natural and Biomedical Polymer Materials, and College of Materials Science and Engineering, Guilin University of Technology, Guilin 541004, China; 2120220300@glut.edu.cn (Z.G.); 1020220174@glut.edu.cn (J.L.); 2 College of Chemistry and Bioengineering, Guilin University of Technology, Guilin 541004, China

**Keywords:** selenium quantum dots, Cr(VI) detection, cell imaging, inner filter effect, intracellular detection

## Abstract

In this paper, we present a simple solvothermal method to synthesize highly fluorescent metal-free elemental selenium quantum dots (SeQDs) using cost-effective bulk selenium powder. The SeQDs exhibit a small and uniform size, excellent aqueous dispersibility, a high photoluminescence quantum yield (PLQY) of 19.3% with stable fluorescence, and scalable production with a 7.2% yield. Owing to the inner filter effect (IFE), these SeQDs function as a highly effective nanoprobe for Cr(VI) detection, exhibiting exceptional sensitivity (detection limit: 145 nM) and selectivity over a wide linear range (5–105 μM), along with rapid response kinetics. Moreover, SeQDs show low cytotoxicity and efficient cellular uptake, enabling cell imaging and intracellular Cr(VI) monitoring. Significant fluorescence quenching in Cr(VI)-exposed cells confirms the potential of SeQDs as a viable fluorescent nanoprobe for Cr(VI) detection in complex cellular environments. This work thus not only establishes a simple method for the preparation of fluorescent SeQDs but also develops a promising fluorescent nanoprobe for cell imaging and Cr(VI) sensing.

## 1. Introduction

The exponential growth of industrial activities has led to a sharp increase in heavy metal contamination, with hexavalent chromium (Cr(VI)) emerging as one of the most harmful pollutants. Chromium exists in various states, with trivalent chromium (Cr(III)) and Cr(VI) being the most stable forms. Cr(IV) is a reductive intermediate of Cr(VI). Generally, Cr(VI) is more toxic than Cr(III) and has severe toxic and carcinogenic effects on living organisms. Even at trace levels, Cr(VI) poses significant risks to both ecological systems and human health, manifesting in dermatological disorders, respiratory impairments, and potential carcinogenicity [1,2]. The sources of Cr(VI) pollution are diverse, with the tannery, electroplating, and textile industries being primary contributors [3]. Given these threats, the development of rapid, cost-effective, and user-friendly analytical methods for the detection of Cr(VI) in aqueous environments is of critical importance. Conventional analytical techniques, such as atomic absorption spectroscopy and inductively coupled plasma mass spectrometry (ICP-MS), offer high precision but are often limited by expensive instrumentation and complex sample pretreatment requirements [4,5]. In contrast, fluorescence-based sensing has garnered significant attention as a viable alternative, combining operational simplicity, a rapid response, and high sensitivity [6,7,8,9]. However, existing fluorescent probes often encounter several key challenges, including the use of toxic precursors during synthesis, limited detection ranges, vulnerability to interference, and complex fabrication processes [10,11,12]. Consequently, the development of an easily accessible and highly effective fluorescent probe for Cr(VI) detection is of paramount significance, both for environmental monitoring and public health protection.

Fluorescent quantum dots (QDs), celebrated for their exceptional photoluminescence, have become attractive tools in sensing and bioimaging applications, thanks to their unique optical characteristics [13,14,15]. However, conventional QDs that incorporate toxic heavy metals (e.g., Cd, Pb) face significant challenges, including cytotoxic risks from ion leaching, environmental hazards during synthesis, and secondary pollution during disposal [16,17]. These limitations highlight the urgent need for biocompatible, metal-free alternatives. Selenium (Se), a non-metallic element with chemical similarities to carbon and sulfur, has emerged as a promising candidate for the synthesis of eco-friendly QDs [18,19]. Its intrinsic biocompatibility, rooted in its role as an essential micronutrient in human physiology, makes it an attractive choice for the development of safer QDs [20,21]. Despite its potential, the current Se-based QDs often incorporate toxic heavy metals (e.g., CdSe, PbSe), which undermine their biosafety and environmental sustainability. Thus, the development of metal-free elemental selenium quantum dots (SeQDs) remains a critical unmet need in the field. The current methods for the synthesis of SeQDs, including laser ablation [22], chemical oxidation, reduction [23,24], and sonochemical exfoliation [25,26], are often hindered by several key challenges. These include low yields (<5%), inadequate photoluminescence quantum yields (PLQY, <10%), complex synthesis procedures, reliance on high-cost raw materials (e.g., NbSe_2_), and stringent equipment requirements. These limitations collectively impede the scalable synthesis and practical application of SeQDs. Therefore, there is a pressing need for simple yet effective strategies to synthesize SeQDs that can overcome these challenges.

**Scheme 1:**
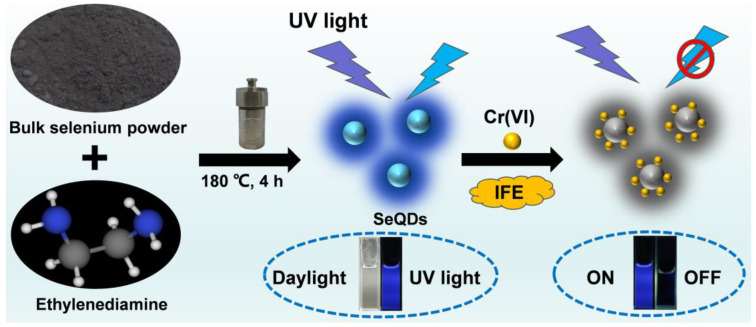
Schematic illustration of preparation of fluorescent SeQDs for sensing of Cr(VI) based on IFE mechanism.

In this study, we demonstrate a simple and viable method for the synthesis of metal-free fluorescent SeQDs through a one-pot solvothermal process using cost-effective bulk selenium (Scheme 1). The resulting SeQDs exhibit a small and uniform size, exceptional aqueous dispersibility, and a high PLQY of 19.3% with stable fluorescence. Crucially, their photoluminescence excitation band exhibits spectral overlap with Cr(VI) absorption bands, enabling sensitive and selective fluorescence quenching via the inner filter effect (IFE) mechanism [27]. This spectral synergy underpins their utility as a sensitive and selective nanoprobe for Cr(VI) detection, achieving a low detection limit with negligible interference from competing metal ions. Beyond aqueous sensing, the SeQDs demonstrate low cytotoxicity and efficient cellular uptake. Their application for intracellular Cr(VI) monitoring was investigated. Given their simple synthesis, superior optical performance, and outstanding sensing capabilities for Cr(VI), this work not only establishes a facile method for the preparation of robust SeQDs but also develops a promising fluorescent nanoprobe for Cr(VI) sensing.

## 2. Materials and Methods

### 2.1. Materials and Chemicals

Bulk selenium powder (≥99.99%, 200 mesh), ethylenediamine (EDA, ≥99.5%), potassium dichromate (K_2_Cr_2_O_7_, ≥99.8%), thiazolyl blue tetrazolium bromide (MTT), iron(III) chloride hexahydrate (FeCl_3_·6H_2_O, ≥99.9%), copper(II) chloride dihydrate (CuCl_2_·2H_2_O, ≥99.8%), sodium chloride (NaCl, 99.99%), nickel(II) nitrate hexahydrate (Ni(NO_3_)_2_·6H_2_O, ≥99.99%), zinc nitrate hexahydrate (Zn(NO_3_)_2_·6H_2_O, ≥99%), calcium chloride (CaCl_2_, 99.99%), potassium chloride (KCl, 99.99%), manganese(II) chloride tetrahydrate (MnCl_2_·4H_2_O, ≥99.8%), cadmium chloride (CdCl_2_, 99.99%), magnesium sulfate (MgSO_4_, 99.99%), aluminum nitrate nonahydrate (Al(NO_3_)_3_·9H_2_O, 99%), mercury(II) nitrate solution (Hg(NO_3_)_2_, 99.99%), and chromium(III) nitrate nonahydrate (Cr(NO_3_)_3_·9H_2_O, 99.99%) were purchased from Beijing Innochem Technology Co., Ltd. (Beijing, China) and used as received. Deionized water was employed throughout the experiments.

### 2.2. Characterization

Transmission electron microscopy (TEM) images were captured with a JEOL-2010 microscope (JEOL Ltd., Tokyo, Japan) at 200 kV. Samples were created by placing SeQD aqueous dispersions on copper grids. Raman spectra came from a LabRam-1B spectroscope (HORIBA Jobin Yvon, Paris, France) with 532.05 nm light. Fourier transform infrared (FTIR) spectra were gathered on a PE Paragon 1000 spectrometer (PerkinElmer, Waltham, MA, USA) via KBr disks. A UV-3600 UV–vis spectrophotometer (Shimadzu, Kyoto, Japan) analyzed the absorption spectra. A Varian Cary 100 spectrometer (Agilent, California, CA, USA) obtained the photoluminescence emission and excitation spectra. A FluoroMax-4 (HORIBA Jobin Yvon, Paris, France) with an integrating sphere measured the photoluminescence quantum yields (PLQY) of the SeQDs and fluorescence decay curves. Cell imaging was performed on a confocal laser-scanning microscope (CLSM, Olympus FV3000) (Olympus, Tokyo, Japan).

### 2.3. Preparation of Fluorescent SeQDs

The fluorescent SeQDs were synthesized using a facile one-pot solvothermal method. In a typical procedure, 55 mg of bulk selenium powder was mixed with 10 mL of EDA in a Teflon-lined autoclave. The mixture was then heated at 180 °C for 4 h. Once the reaction was complete, the dark brown mixture was transferred to a round-bottom flask and subjected to rotary evaporation under reduced pressure at 80 °C to eliminate the EDA. After evaporation, 20 mL of ethanol was added to the residue and stirred thoroughly. The resulting SeQD precipitates were collected by centrifugation at 10,000 rpm for 5 min and washed repeatedly with ethanol. Finally, the SeQDs were dispersed in water and stored at 4 °C for subsequent detection applications. A portion of the SeQDs was dried for structural characterization.

### 2.4. Sensing of Cr(VI)

The fluorescence-based detection of Cr(VI) was performed using an aqueous dispersion of SeQDs under ambient conditions. In a standard assay, 0.6 mL of the SeQD (1 mg/mL) dispersion was transferred into a quartz cuvette, followed by the incremental addition of Cr(VI) standard solutions (or other analytes) at predetermined concentrations. The mixture was diluted to a final volume of 3 mL with deionized water, homogenized via brief vortexing (60 s), and subjected to fluorescence spectral analysis using a fixed excitation wavelength of 340 nm.

### 2.5. Cytotoxicity Evaluation

The cytotoxic effects of SeQDs were assessed via the MTT assay. B16 melanoma and L929 fibroblast cells were cultured in 96-well plates at 1 × 10^4^ cells/mL. After 24 h, the medium was replaced with SeQD dispersions (0–200 μg/mL); then, the cells were further incubated for 24 h. The wells were rinsed thrice with PBS; then, 100 μL of fresh 0.5 mg/mL MTT solution was added to the culture medium. After 3 h, the MTT medium was discarded and 100 μL dimethyl sulfoxide was added per well, with gentle shaking for 20 min. Absorbance at 490 nm was measured using a microplate reader. Cell viability was determined by the ratio of the absorbance in SeQD-treated wells to that in untreated controls.

### 2.6. Cell Imaging and Monitoring of Cr(VI) in Cells

The B16 and L929 cell lines were maintained in Dulbecco’s modified Eagle’s medium (DMEM) under standard culture conditions (37 °C, 5% CO_2_) for 12 h. Following medium removal, the cells were exposed to SeQD-supplemented DMEM (200 μg/mL) for 1 h under identical conditions. Unbound SeQDs were eliminated via three sequential washes with phosphate-buffered saline (PBS), after which the intracellular fluorescence was visualized using CLSM. To evaluate Cr(VI) detection in cells, SeQD-labeled cells were incubated with Cr(VI) solution (50 μM). Post-treatment fluorescence imaging was performed immediately using CLSM to monitor the fluorescence quenching kinetics.

## 3. Results and Discussion

### 3.1. Preparation and Characterization of SeQDs

As illustrated in Scheme 1, fluorescent SeQDs could be efficiently prepared from bulk Se powder and ethylenediamine (EDA) through solvothermal treatment at 180 °C for 4 h. This method yielded a product with notably different properties compared to the solvothermal treatment of EDA alone under identical conditions, which produced only a minimal amount of ethanol-soluble viscous material. In contrast, the SeQDs exhibited extremely limited dispersibility in ethanol (about 0.03 mg/mL), enabling simple purification through ethanol precipitation. The yield of SeQDs achieved through our approach was determined to be 7.2%, significantly outperforming other reported methods. The resulting SeQDs demonstrated excellent water dispersibility and emitted intense blue fluorescence under 365 nm UV light illumination.

**Figure 1:**
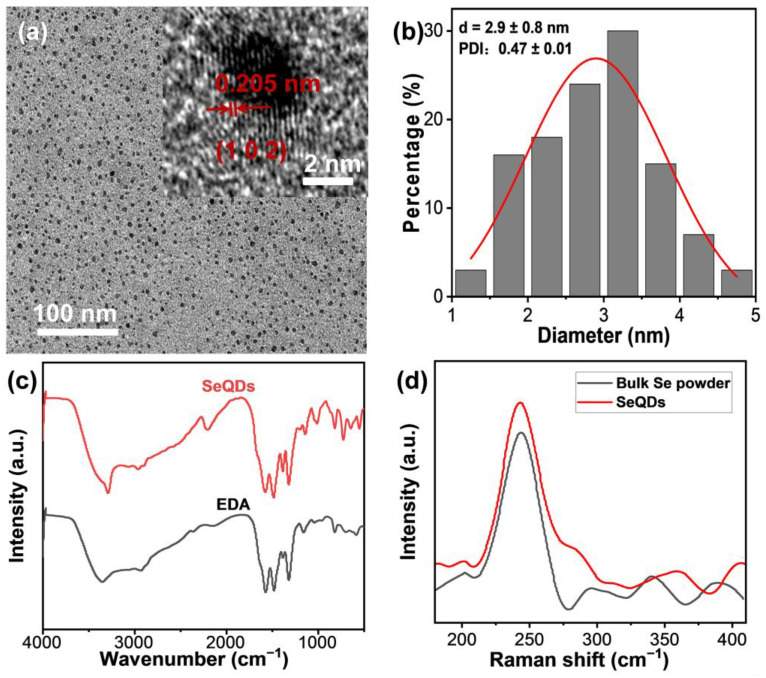
(**a**) Representative TEM and high-resolution TEM (inset) images of SeQDs. (**b**) Size distribution of SeQDs. (**c**) FTIR spectra of EDA and SeQDs. (**d**) Raman spectra of bulk Se powder and SeQDs.

Morphological and structural characterization via transmission electron microscopy (TEM) revealed that the SeQDs possessed a uniform quasi-spherical morphology with an average diameter of approximately 2.9 nm (Figure 1a,b). The diameter of the SeQDs was determined by measuring one hundred particles, which were then categorized into eight groups based on Sturges’ Rule [28]. Subsequently, Gaussian fitting was applied to process the data. The high-resolution TEM (HRTEM) image further confirmed the crystalline nature of the SeQDs, displaying distinct lattice fringes with spacing of 0.205 nm, which corresponded to the (102) crystal plane (as shown in the inset of Figure 1a). This observation is consistent with previously reported SeQDs [25]. Chemical analysis through Fourier-transform infrared (FTIR) spectroscopy identified characteristic peaks at 3304 cm^−1^ (N-H stretching vibrations) and 1327 cm^−1^ (C-N stretching vibrations) in the SeQD spectrum (Figure 1c). Upon comparison of the FTIR results, it was evident that the characteristic peaks of EDA and the SeQDs showed no significant differences. This observation is likely due to the chelating effect of EDA. Following the reaction, EDA molecules anchor onto the surfaces of the SeQDs, acting as a ligand. This action enhances the aqueous stability and dispersibility of the SeQDs. Raman spectroscopy provided additional confirmation of the SeQDs’ composition, with a prominent peak at 245 cm^−1^ corresponding to Se-Se bond vibrations [23] (Figure 1d), matching the reference spectra of the bulk Se powder. Collectively, these comprehensive characterizations confirmed the successful synthesis of fluorescent SeQDs, establishing a robust foundation for their application in Cr(VI) detection.

### 3.2. Optical Properties and Cytotoxicity Evaluation of SeQDs

**Figure 2:**
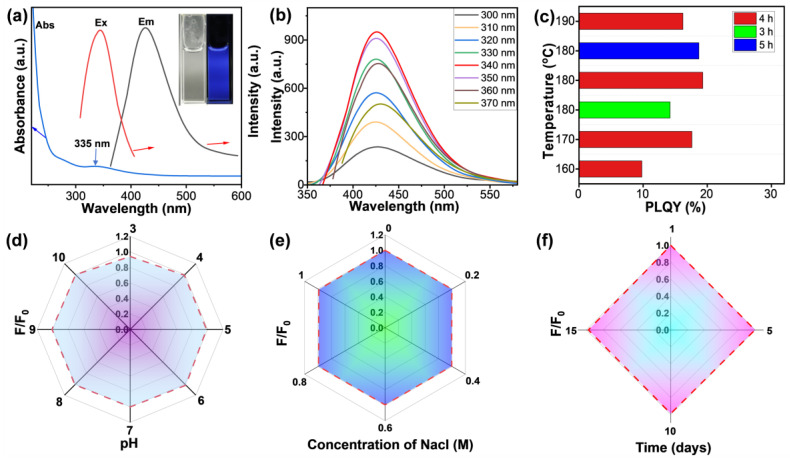
(**a**) UV–vis absorption (Abs), photoluminescence excitation (Ex), and emission (Em) spectra of SeQD dispersions. Inset: Photographs of SeQD aqueous dispersions under daylight and 365 nm UV illumination. (**b**) Emission spectra of SeQD aqueous dispersions at various excitation wavelengths. (**c**) PLQYs of SeQDs synthesized at different temperatures and reaction times. Effects of pH (**d**), NaCl concentration, and (**e**) storage time (**f**) on relative fluorescence intensity (F/F_0_) of SeQDs.

The photophysical characteristics of the SeQDs in aqueous dispersions were comprehensively analyzed through UV–vis absorption and photoluminescence spectroscopy (Figure 2a). The absorption spectrum displays a broad feature centered at 335 nm, attributed to synergistic contributions from n→π* electronic transitions in non-bonding orbitals and interband transitions within zero-valent selenium. Notably, the SeQDs exhibit excitation-independent emission, maintaining a fixed emission maximum at 425 nm across excitation wavelengths spanning 300–370 nm (Figure 2b). This 425 nm emission band in the SQDs is primarily due to the intrinsic photoluminescence of the quantum dots, influenced by their size, surface states, and reaction conditions. The optimal fluorescence intensity was achieved under 340 nm excitation, correlating with a distinct excitation peak at 340 nm in the photoluminescence excitation spectrum (Figure 2a). The synthesis parameters were systematically optimized by varying the reaction temperatures (160–190 °C) and durations (3–5 h). The highest PLQY of 19.3%—surpassing that of most reported SeQDs—was achieved at 180 °C over 4 h (Figure 2c). Remarkably, even at an ultralow concentration of 0.04 mg/mL, the colorless aqueous dispersion of SeQDs emitted intense blue fluorescence under 365 nm UV light (inset of Figure 2a). The SeQDs also demonstrated exceptional environmental stability. The fluorescence intensity remained stable across acidic to alkaline conditions (pH 3–10, Figure 2d), under high ionic strength (1 M NaCl, Figure 2e), and during prolonged storage (15 days, Figure 2f). These high PLQY and robust fluorescence properties render the SeQDs highly promising for sensing applications in complex environments.

**Figure 3:**
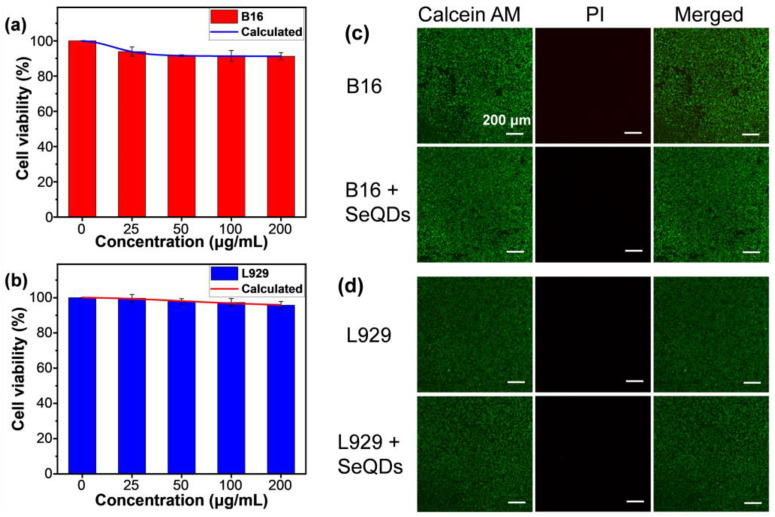
Cell viability of B16 cells (**a**) and L929 cells (**b**) after incubation with SeQDs for 24 h. CLSM images of B16 cells (**c**) and L929 cells (**d**), both with and without incubation of SeQDs, which were co-stained using calcein AM for live cell staining and PI for dead cell staining.

The biocompatibility of the SeQDs was thoroughly evaluated to determine their applicability. Cytotoxicity assays using the thiazolyl blue tetrazolium bromide (MTT) assay on B16 mouse melanoma cells and L929 mouse fibroblast cells demonstrated remarkable cellular tolerance. Both cell lines maintained over 91% viability after 24 h exposure to the SeQDs at concentrations up to 200 µg/mL (Figure 3a,b). The MTT assay data were fitted using the Hill equation [29], revealing that the SeQDs exhibited high biocompatibility with both cell lines. In addition, a live/dead dual-fluorescence assay was conducted using calcein AM (green fluorescence in live cells) and propidium iodide (PI, red fluorescence in dead cells). The confocal laser scanning microscopy (CLSM) images revealed predominantly green fluorescence in both cell types treated with 200 µg/mL SeQDs (Figure 3c,d), with no detectable PI signal. This outcome confirms the negligible cell mortality and aligns with the MTT results. These results collectively demonstrate the low cytotoxicity of the SeQDs, highlighting their potential for practical applications.

### 3.3. Detection of Cr(VI) by SeQDs

**Figure 4:**
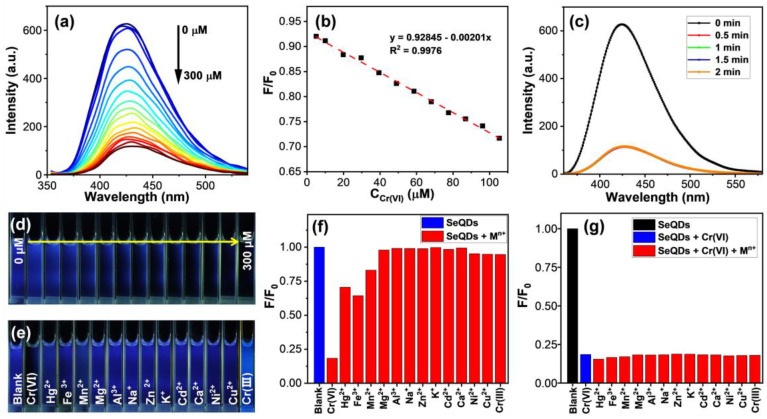
(**a**) Emission spectra of SeQDs upon gradual addition of Cr(VI) (λ_ex_ = 340 nm). (**b**) F/F_0_ value of SeQDs in response to different concentrations of Cr(VI) in the range of 5–105 µM. (**c**) Emission spectra of SeQDs after mixing with Cr(VI) (300 µM) for different times. Photographs of SeQD aqueous dispersions after mixing with different concentrations of Cr(VI) (0–300 µM) (**d**) or various metal ions (300 µM) (**e**) under 365 nm UV lamp. (**f**) F/F_0_ values of SeQD dispersions in the presence of 300 µM diverse metal ions. (**g**) F/F_0_ values against 300 μM of Cr(VI) (blue column) and 300 μM of other metal ions together with 300 μM of Cr(VI) (red columns). F_0_ and F represent the fluorescence intensity of SeQDs in the absence and presence of Cr(VI) or/and other metal ions, respectively.

To explore the feasibility of SeQDs as a fluorescent nanosensor for the sensing of Cr(VI), the fluorescence emission spectra of the SeQDs were recorded across varying Cr(VI) concentrations (0–300 μM). As presented in Figure 4a, the SeQDs’ fluorescent intensity at 425 nm decreased significantly with increasing Cr(VI) concentrations, indicating high sensitivity to Cr(VI). Specifically, the fluorescence intensity dropped by 81.6% at 300 μM Cr(VI). The quenching efficiency (F/F_0_) shows a linear relationship with the Cr(VI) concentration ranging from 5 to 105 μM (Figure 4b), with a correlation coefficient of 0.9976. The standard deviation (SD) of the residuals from the linear fit was determined to be 0.003. This linear correlation enables the quantitative detection of Cr(VI) with a limit of detection (LOD) of 145 nM (equivalent to 7.5 μg/L). The LOD for Cr(VI) was calculated using the formula

LOD = 3σ/k(1)

Here, σ represents the standard deviation of the blank sample (with n = 5 measurements), and k denotes the slope of the linear calibration curve. This sensitivity is sufficient to detect Cr(VI) in drinking water, as it meets the stringent standard of 50 μg/L set by the US Environmental Protection Agency [30]. Additionally, the sensing performance of SeQDs for Cr(VI) detection is highly competitive and surpasses that of many existing fluorescent probes [31,32,33,34,35,36,37] (Table 1). Furthermore, the fluorescence quenching process is rapid, completing within 0.5 min (Figure 4c). As can be seen in Figure 4d, the blue fluorescence of the SeQD aqueous dispersions was weakened with increasing Cr(VI) concentrations, with no detectable fluorescence at 300 μM Cr(VI). These results establish SeQDs as a sensitive, rapid-response platform for Cr(VI) detection in aqueous environments.

To assess the selectivity of SeQDs for Cr(VI) detection, fluorescence quenching assays were performed in the presence of various metal ions (Hg^2+^, Fe^3+^, Mn^2+^, Mg^2+^, Al^3+^, Na^+^, Zn^2+^, K^+^, Cd^2+^, Ca^2+^, Ni^2+^, Cu^2+^, and Cr(III)) at a concentration of 300 μM. As illustrated in Figure 4e, under 365 nm UV light, the SeQD dispersion exhibited remarkable quenching in response to Cr(VI), while other metal ions caused negligible (e.g., Mg^2+^, Al^3+^, and Na^+^,) or only slight (e.g., Hg^2+^, Fe^3+^, and Cr(III)) quenching effects. The quantitative analysis (Figure 4f) confirmed that Cr(VI) induced a significant reduction in the fluorescence intensity, while other ions had a minimal impact, highlighting the exceptional selectivity of SeQDs for Cr(VI). Even in multicomponent systems containing equimolar concentrations of interfering metal ions (Figure 4g), Cr(VI)-specific quenching remained dominant. These results validate the SeQDs as a robust and interference-resistant nanoprobe for the selective sensing of Cr(VI).

**Figure 5:**
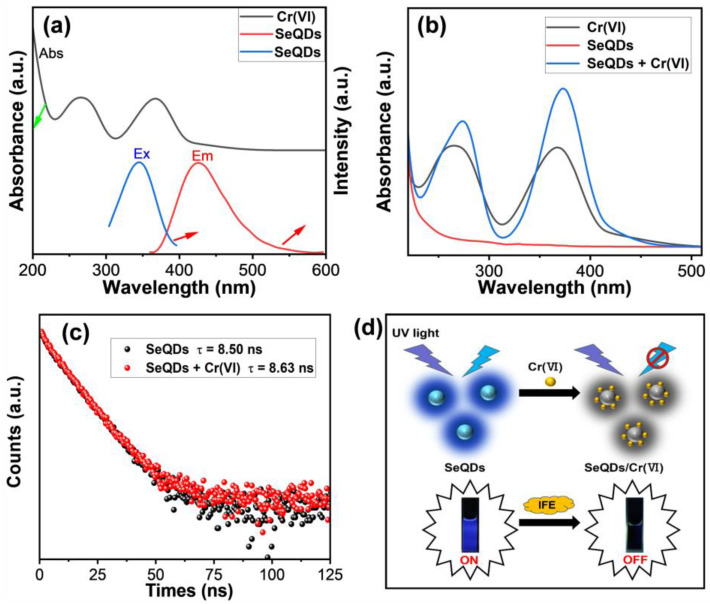
(**a**) UV–vis absorption spectrum of Cr(VI) and photoluminescence excitation and emission spectra of SeQDs. (**b**) UV–vis absorption spectra of Cr(VI), SeQDs, and their mixture. (**c**) Time-resolved fluorescence decay curves of SeQDs before and after mixing with Cr(VI) (300 µM). (**d**) Schematic illustration of the fluorescence detection mechanism for Cr(VI) using SeQDs.

To elucidate the quenching mechanism involving Cr(VI), the UV–vis absorption spectrum of a Cr(VI) solution was measured. As depicted in Figure 5a, the absorption spectrum of Cr(VI) exhibits significant overlap with the excitation band of SeQDs and partial overlap with their emission band, implying potential fluorescence quenching mechanisms involving either the IFE or Förster resonance energy transfer (FRET) [38]. To elucidate the dominant mechanism, the UV–vis absorption spectra were compared for Cr(VI), the SeQDs, and their mixture. The SeQD/Cr(VI) mixture exhibited absorption profiles similar to those of Cr(VI) alone, indicating no complex formation between the SeQDs and Cr(VI) (Figure 5b). Furthermore, fluorescence lifetime measurements revealed minimal changes between pristine SeQDs (8.50 ns) and those mixed with Cr(VI) solution (8.63 ns), as illustrated in Figure 5c. This finding confirms that the IFE is the dominant mechanism behind fluorescence quenching, rather than FRET. Energy transfer processes typically induce substantial changes in fluorescence lifetimes, which were not observed here. Thus, the observed fluorescence quenching is attributed to the IFE, where Cr(VI) acts as an optical filter, absorbing the excitation energy and blocking the emission light of the SeQDs (Figure 5d).

**Figure 6:**
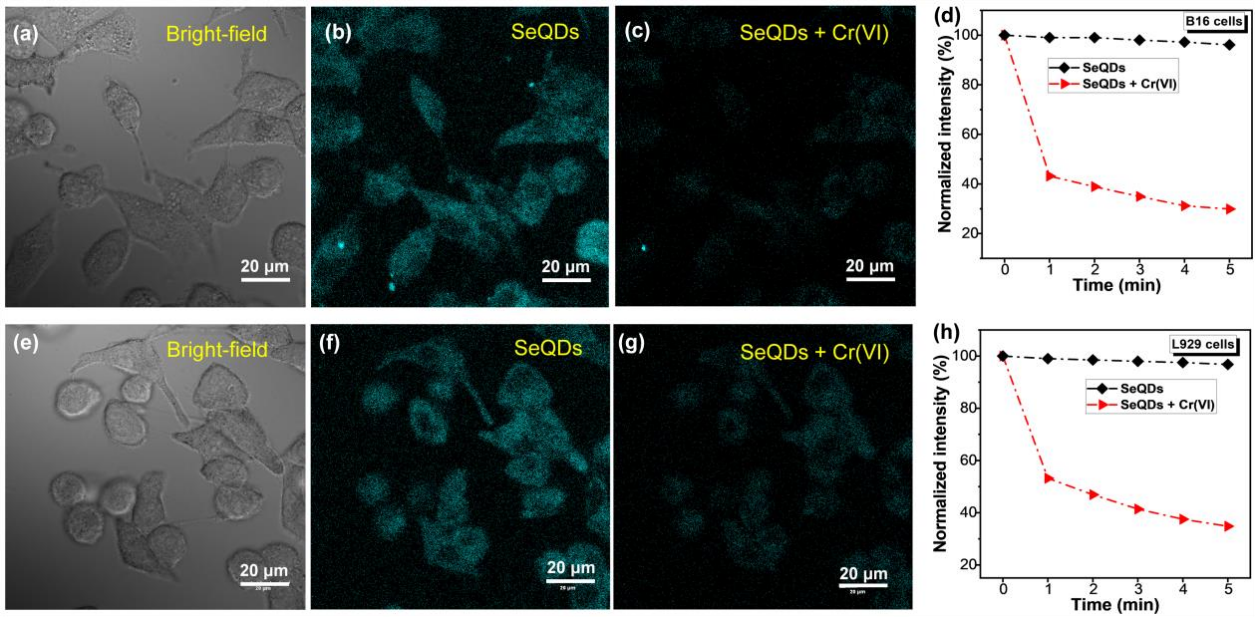
(**a**) CLSM bright-field image of SeQD-stained B16 cells. CLSM fluorescence images of SeQD-stained B16 cells before (**b**) and after (**c**) incubation with Cr(VI) solution (50 µM) for 1 min. (**d**) Comparison of the fluorescence intensity of SeQD-stained B16 cells with and without incubation in Cr(VI) solution for various durations. (**e**) CLSM bright-field image of SeQD-stained L929 cells. CLSM fluorescence images of SeQD-stained L929 cells before (**f**) and after (**g**) incubation with Cr(VI) solution (50 µM) for 1 min. (**h**) Comparison of the fluorescence intensity of SeQD-stained L929 cells with and without incubation in Cr(VI) solution for various durations.

The low cytotoxic potential and distinctive fluorescence sensitivity of SeQDs toward Cr(VI) motivated their investigation for cellular imaging and intracellular Cr(VI) monitoring (Figure 6). The CLSM imaging of B16 and L929 cells following 1 h incubation with SeQDs revealed distinct blue emission signals under 405 nm excitation (Figure 6b,f), confirming the successful cellular internalization of the SeQDs. The subsequent exposure of SeQDs-labeled cells to a 50 µM Cr(VI) solution induced rapid fluorescence diminution, with the signal intensity becoming markedly attenuated within 1 min of treatment (Figure 6c,g). The quantitative analysis of the integrated fluorescence intensities demonstrated statistically significant reductions in cellular emission as the Cr(VI) exposure duration increased, compared to the SeQD-labeled controls (Figure 6d,h). These findings validate the capability of SeQDs to function as an effective fluorescent nanoprobe for the sensing of Cr(VI) in intricate biological systems.

## 4. Conclusions

In summary, this study achieves obvious progress in developing metal-free SeQDs via a simple and scalable solvothermal synthesis method using low-cost bulk selenium powder. The SeQDs exhibit a small and uniform size, excellent aqueous dispersibility, a high PLQY, and stable fluorescence in complex environments. They demonstrate sensitive, rapid, and selective fluorescence quenching toward Cr(VI) through the IFE mechanism, achieving a low detection limit and minimal interference from competing metal ions. Moreover, the SeQDs show low cytotoxicity and efficient cellular uptake, enabling intracellular Cr(VI) monitoring through significant fluorescence attenuation in cells. This work not only advances material innovation by providing a simple and scalable synthesis route for robust SeQDs, but also establishes a highly promising fluorescent nanoprobe for cell imaging and Cr(VI) tracking in environmental and biological systems.

## Figures and Tables

**Table 1:** Comparison of diverse fluorescent probes for sensing of Cr(VI).

Fluorescent Probe	Linear Range	LOD	Reference
S, N-CDs	0.1–50 µM	92 nM	[31]
His-Cu NCs	0–60 µM	310 nM	[32]
Nitrogen-rich CQDs	5–100 µM	700 nM	[33]
Fe-doping CDs	0.25–100 µM	150 nM	[34]
CS-CDs	10–300 µM	2830 nM	[35]
ZnO QDs	1–17.5 µM	1130 nM	[36]
D-CDs	0.5–400 µM	80 nM	[37]
This work	5–105 μM	145 nM	This study

## Data Availability

The data presented in this study are available on request from the corresponding author due to due to privacy.
